# Interactions of Antibiotics and Methanolic Crude Extracts of *Afzelia Africana* (Smith.) Against Drug Resistance Bacterial Isolates

**DOI:** 10.3390/ijms12074477

**Published:** 2011-07-13

**Authors:** Olayinka Aiyegoro, Adekanmi Adewusi, Sunday Oyedemi, David Akinpelu, Anthony Okoh

**Affiliations:** 1 Department of Pharmaceutical Chemistry, North-West University, Potchefstroom 2520, South Africa; 2 Department of Biochemistry and Microbiology, University of Fort Hare, Alice 5700, South Africa; E-Mail: aokoh@ufh.ac.za; 3 Department of Pharmacology, Faculty of Health Sciences, University of Pretoria, Pretoria 0001, South Africa; E-Mail: adewusiadekanmi@gmail.com; 4 Department of Botany, University of Fort Hare, Alice 5700, South Africa; E-Mail: silvanusdemi@gmail.com; 5 Department of Microbiology Obafemi Awolowo University, Ile Ife 23436, Nigeria; E-Mail: dakinpel@oauife.edu.ng

**Keywords:** *Afzelia Africana*, synergy, antibiotics, extract, drug-resistant, microorganisms

## Abstract

Infection due to multidrug resistance pathogens is difficult to manage due to bacterial virulence factors and because of a relatively limited choice of antimicrobial agents. Thus, it is imperative to discover fresh antimicrobials or new practices that are effective for the treatment of infectious diseases caused by drug-resistant microorganisms. The objective of this experiment is to investigate for synergistic outcomes when crude methanolic extract of the stem bark of *Afzelia africana* and antibiotics were combined against a panel of antibiotic resistant bacterial strains that have been implicated in infections. Standard microbiological protocols were used to determine the minimum inhibitory concentrations (MICs) of the extract and antibiotics, as well as to investigate the effect of combinations of the methanolic extract of *A. africana* stem bark and selected antibiotics using the time-kill assay method. The extract of *Afzelia africana* exhibited antibacterial activities against both Gram-negative and Gram-positive bacteria made up of environmental and standard strains at a screening concentration of 5 mg/mL. The MICs of the crude extracts and the antibiotics varied between 1 μg/mL and 5.0 mg/mL. Overall, synergistic response constituted about 63.79% of all manner of combinations of extract and antibiotics against all test organisms; antagonism was not detected among the 176 tests carried out. The extract from *A. africana* stem bark showed potentials of synergy in combination with antibiotics against strains of pathogenic bacteria. The detection of synergy between the extract and antibiotics demonstrates the potential of this plant as a source of antibiotic resistance modulating compounds.

## 1. Introduction

The ample exploit of antibiotics in the management of bacterial infections has led to the emergence and multiplication of resistant bacterial strains. Infection due to multidrug resistance pathogens are routinely complicated to deal with due to virulence factors and because of a relatively limited choice of antimicrobial agents. Thus, it is extremely important to find novel antimicrobials or new techniques that are effective for the treatment of infectious diseases caused by drug-resistant microorganisms. Two drugs used in mixture may yield enhanced or reduced end product, combinations of two drugs, that yield visibly similar effects may produce synergistic or antagonistic interactions. Few studies have found that the efficacy of antimicrobial agents can be improved by combining them with crude plant extracts against different pathogens and a number of compounds with an *in vitro* activity of reducing the minimum inhibitory concentrations (MICs) of antibiotics against resistant organisms have been isolated from plants. Researchers [1–3], have demonstrated that plants either contain antimicrobials that can operate in synergy with antibiotics or posses compounds that have no intrinsic antibacterial activity but are able to sensitize the pathogen to a previously ineffective antibiotic.

*Afzelia africana* belongs to the family Caesalpiniaceae. The English name of the plant is mahogany. The tree is widely distributed in Africa and Asia [4], where it is used as food and for plank wood, as well as widely used as folklore remedies among many tribes in Africa. Previous studies have reported the plant to exhibit anti-inflammatory and analgesic bioactivities [5]. Atawodi [6] reported trypanocidal activities of the leaves and stem bark extract of the plant against *Trypanosoma brucei*. Powdered root of the plant mixed with millet beer has been used as treatment for hernia among some tribes in Cote d’Ivoire [7]. We have also previously reported the antibacterial activity in the crude extract of the stem bark of *Afzelia africana* [8] and the biocidal and cell membrane disruption potentials of stem bark extracts of *A. africana* [9]. In this paper, we reported the effects of combinations of the stem bark extract of *A. africana* and some antibiotics at MICs and Sub MICs levels on their resistance modifying potencies against bacterial pathogens that are implicated in infections.

## 2. Results and Discussion

The extraction gave 100 g for the methanol crude extracts that was brown in color. The results of these experiments revealed that crude methanolic stem bark extract of *Afzelia africana* exhibited antibacterial activities against the test bacterial isolates comprising of both Gram-negative and Gram-positive bacteria made up of environmental and standard strains, at a screening concentration of 5 mg/mL (Table 1). Zones of inhibition ranged from 13–25 mm for the methanol extract. The least activity with an inhibition zone diameter (IZD) of 13 mm was against *Staphylococcus aureus* (ATCC 6538) and the highest antibacterial activity was against environmental strain of *Micrococcus kristinae* with zone of inhibition of 25 mm diameter. The antibiotics, tetracycline and ampicilin yielded zones of inhibition that ranged between 10–28 mm and 13–30 mm respectively. The MICs of the crude extracts and the antibiotics varied between 1 μg/mL and 5.0 mg/mL (Table 2). Specifically, the MICs ranged from 0.1–5.0 mg/mL for the crude methanol extract, the lowest MIC value (0.1 mg/mL) for the extracts was against *K. pneumoniae* (ATCC 10031) and environmental strain of *P. vulgaris* while the highest value (5.0 mg/mL) was against *P. vulgaris* (CSIR 0030) and environmental strain of *K. pneumoniae*. For the standard antibiotics, the ranges were 0.001–0.412 mg/mL for penicillin G, erythromycin and amoxicillin; 0.001 to 0.004 mg/mL for ciprofloxacin and ampicilin; 0.001–0.256 mg/mL for chloramphenicol; 0.001–0.032 mg/mL for oxytetracycline and tetracycline (Table 2). The time kill effect of combinations of the methanol extract of *A. africana* stem bark and antibiotics is shown in Table 3 and 4. The extract showed ability to improve the bactericidal effect of the antibiotics on both Gram-positive and Gram-negative microorganisms. The highest bactericidal activity exemplified by a 4.52 ± 0.09. Log_10_ reduction in cell density was observed against *Micrococcus luteus* (environmental strain) when the extract and Chloramphenicol are combined at MIC values. At 1/2MIC level, 59.09% synergy; 40.91% indifference and no antagonism were observed; and at MIC level, 67.05% synergy; 32.95% and no antagonism were observed. Overall, synergistic response constituted about 63.79% of all manner of combinations of extract and antibiotics against all test organisms; antagonism was not detected among the 176 tests carried out.

To evaluate the outcome of combinations between the extracts of the plant and antibiotics, the MIC values of the antibiotics was determined as these provide the reference point for defining the interactions. The aim of testing the plant extracts for probability of synergy with antibiotics is to explore if mixture of such extracts with antibiotics can bring about positive alteration in the vulnerability of the test strains, thus necessitating the use of strains resistant to the test antibiotics. Therefore, the British Society for Antimicrobial Chemotherapy [10], recommend that MIC breakpoints were used. Although this data is often used in surveillance studies to monitor trends in resistance development, we deemed it convenient to apply in our studies in the absence of a standard. According to the MIC breakpoints, strains of *Staphylococcus aureus and Enterococcus faecalis* with MIC values of ≥0.25 mg L^−1^ (for penicillin G), ≥2 mg L^−1^ (for amoxicillin), ≥2 mg L^−1^ (for tetracycline), ≥1 mg L^−1^ (for erythromycin), ≥4 mg L^−1^ (for chloramphenicol) and ≥1 mg L^−1^ (for ciprofloxacin) are classified as resistant.

From results of the MIC values obtained (Table 2), it is obvious that almost all of the bacteria tested were resistant or just slightly susceptible to the test antibiotics [10]. The MIC values for these organisms ranged from 1 to 512 times higher than the predicted breakpoint values. The breakpoint values for enteric bacteria are; 16 mg L^−1^ (penicillins), 2 mg L^−1^ (tetracycline), 16 mg L^−1^ (chloramphenicol) and 1 mg L^−1^ (ciprofloxacin) [10].

*A. africana* crude stem bark extract inhibited the growth of all the test organisms (Tables 1 and 2) that have been implicated in infections. This finding supports the use of *A. africana* in the treatment of diseases caused by these pathogens. The antimicrobial activity of *A. africana* extract successfully inhibited the growth of both Gram-positive and Gram-negative bacteria isolates used in this study, thus, exhibiting a broad spectrum of activities. Combinations of some herbal materials and different antibiotics might affect the inhibitory effect of these antibiotics [17]. Such combinations would be synergistic if there is a decrease in the MIC of each agent of four-fold; partially synergistic if there is a MIC decrease for one drug of four-fold and a decrease of two-fold of the other agent; additive if there is a two-fold reduction in the MIC of both agents; indifference is all interactions not meeting the criteria listed above and not being antagonistic. Antagonistic response refers to where a MIC increase of four-fold for each drug would be observed in combination [18]. The time-kill assay detected synergy against both Gram-positive and Gram-negative bacteria. The synergy detected in this study was not specific to any group of organisms or class of antibiotics. This suggests that crude extracts of this plant could contain a mixture of compounds that can enhance the activity of different antibiotics.

*A. africana* is known to contain a number of antimicrobial compounds [19], such as polyphenols and flavonoids. Thus natural products and phytocompounds can both be exploited for novel bioactive compounds or may be used in the development of standardized herbal medicine to control infectious diseases.

The antimicrobial and resistance modifying potentials of naturally occurring compounds have been reported in studies, such as Sato *et al.* and Cushnie and Lamb [17,20]. This would suggest that the synergy with antibiotics observed in this study could be attributable to such compounds. Some of these compounds, like polyphenols, have been shown to exert their antibacterial action through membrane perturbations. This perturbation of the cell membrane coupled with the action of β-lactams on the transpeptidation of the cell membrane could lead to an enhanced antimicrobial effect of the combination [21].

It has also been shown that some plant derived compounds can improve the *in vitro* activity of some peptidoglycan inhibiting antibiotics by directly attacking the same site (*i.e.*, peptidoglycan) in the cell wall [22]. While the above explanations may account for the synergy between the extracts and β-lactam antibiotics that act on the cell wall, it might not apply in the case of the observed synergy with other classes of antibiotics with different targets, such as tetracyclines, erythromycin, ciprofloxacin and chloramphenicol.

Bacterial efflux pumps are responsible for a significant level of resistance to antibiotics in pathogenic bacteria [23]. Some plant-derived compounds have been observed to enhance the activity of antimicrobial compounds by inhibiting MDR efflux systems in bacteria [24]. 5′-methoxyhydnocarpin is an example of an inhibitor of the NorA efflux pump of *Staphylococcus aureus* isolated from *Berberis fremontii* [25]. It is likely that the methanolic stem bark extract of *A. africana* could contain potential efflux pump inhibitors. Such compounds are likely to be broad spectrum efflux inhibitors considering that the synergistic effect of the extract was observed on both Gram-positive and Gram-negative bacteria, as well as in combination with cell wall inhibiting and protein synthesis inhibiting antibiotics. In fact, some broad spectrum efflux pump inhibitors have been isolated from some plants. Smith [26] reported one efflux inhibitor (ferruginol) from the cones of *Chamaecyparis lawso-niana*, which inhibited the activity of the quinolone resistance pump (NorA), the tetracycline resistance pump, (TetK) and the erythromycin resistance pump, (MsrA) in *Staphylococcus aureus*.

Antibiotics could interfere with bacterial cell wall synthesis, increase bacterial membrane permeability and/or inhibit bacterial protein synthesis at the 30S subunit of ribosomes [27]; therefore the different modes of action of the extract from the antibiotics may be an important factor in the enhanced bactericidal efficacy observed when used in combination.

Since the combinations of extract with antibiotics could inhibit both Gram-positive and Gram negative pathogenic bacteria, and most combinations were synergistic or indifferent, these kinds of combinations may be practical and beneficial in inhibiting both pathogens. Also, the required dosage of these antibiotics used in combination may be less than when used alone, which may further reduce the occurrence of side effects caused by these antimicrobials.

The strong synergy observed between the crude methanolic extract of the stem bark of *A. africana* and firstline antibiotics is a significant finding demonstrating the therapeutic potentials of this plant. However, the synergy pattern between these plant extracts and antibiotics is suspected to depend mainly on genetic compositions and interactive modes of action, rather than concentrations of the two agents in combinations, although, concentrations relative to MIC may have a major role to play in the outcomes of the combinational experiment. However, it must be emphasized, that these findings may be difficult to present as a case, until after the mechanism of the active component(s) of the crude extract has been studied and determined.

## 3. Experimental Section

### 3.1. Materials and Methods

Plant Material and Extract Preparation: Fresh stem bark of *A. africana* used in this study was collected in Abeokuta, Ogun State, Nigeria in the month of April 2008. The stem bark was air-dried to constant weight, powdered and stored in an air-tight container for further use. Exactly 750 g of the powdered bark of *A. africana* was extracted in cold using methanol and sterile distilled water in 3:2 ratios for 4 days. The mixture was then filtered and the filtrate was first concentrated *in vacuo* using rotary evaporator to remove the organic solvent. The remaining aqueous residue was lyophilized to obtain the crude extract. The extract was brown in color and the yield collected was about 100 g.

### 3.2. Preparation of Bacterial Inoculums

Bacterial isolates used in this study included reference strains obtained from the South African Bureau of Standards (SABS); *Enterococcus faecalis* (ATCC 29212), *Staphylococcus aureus* (ATCC 6538), *Bacillus pumilus* (ATCC 14884), *Klebsiella pneumoniae* (ATCC 10031) and *Proteus vulgaris* (CSIR 0030). The environmental strains include *Micrococcus kristinae, Micrococcus luteus*, *Proteus vulgaris*, *Klebsiella pneumoniae*, *Bacillus subtilis*, *Staphylococcus epidermidis*, which were obtained from the Applied and Environmental Microbiology Research Group (AEMREG), Department of Biochemistry and Microbiology, University of Fort Hare, Alice, South Africa. The inoculums of the test organisms were prepared using the colony suspension method [10]. Colonies picked from 24 h old cultures grown on nutrient agar were used to make suspensions of the test organisms in saline solution to give an optical density of approximately 0.1 at 600 nm. The suspension was then diluted 1:100 by transfer of 0.1 mL of the bacterial suspension to 9.9 mL of sterile nutrient broth before use.

### 3.3. Antibiotics Used in this Study

The following antibiotics were used in this study: Penicillin G sodium (Duchefa), Amoxicillin (Duchefa), Chloramphenicol (Duchefa), Oxytetracycline (Duchefa), Ampicilin sodium salt (Calbiochem), Tetracycline hydrochloride (Duchefa), Erythromycin (Duchefa) and Ciprofloxacin (Fluka).

### 3.4. Sensitivity Testing of the Crude Plant Extract

The sensitivity testing of the crude extract of the plant was determined using the agar-well diffusion method as described by [11,12], with modifications. The bacterial isolates were first grown in nutrient broth for 18 h to prepare bacterial suspension as described above. The bacterial suspension (0.1 mL) was inoculated into molten Mueller-Hinton agar medium at 50 °C and then poured into a sterile Petri dish, the plate was allowed to set and wells were then bored into the agar medium using a sterile 6 mm cork borer. The wells were later filled up with about 0.2 mL of the extract at a concentration of 5 mg/mL in total of 5% methanol, taking care to prevent spillage onto the surface of the agar medium. The plates were allowed to stand on the laboratory bench for 1 h to allow proper diffusion of the extract into the medium and then incubated at 37 °C for 24 h, after which they were observed for zones of inhibition. Tetracycline and ampicillin at concentration of 0.1 mg/mL and 10 μg/mL, respectively, were used as controls to compare the effect of the extract to those of the standard antibiotics.

### 3.5. Determination of the Minimum Inhibitory Concentrations (MICs)

The minimum inhibitory concentrations (MICs) of the antibiotics and plant extracts were determined using the standard method of the European Committee for Antimicrobial Susceptibility Testing [10]. Dilutions of the antibiotics, ranging from 0.001–0.824 mg/mL in nutrient agar (Biolab), were prepared by incorporating the antibiotic stock solution into molten agar at 50 °C. Dilutions of the extract ranging from 0.05–20 mg/mL were also prepared and incorporated into molten nutrient agar (Biolab) at 50 °C and poured into sterile plates. The plates were allowed to set and then inoculated with standardized inocula of the test bacteria by streaking on the plate surface. Plates were incubated at 37 °C for 24 h under aerobic conditions. The MIC was defined as the lowest concentration of the antibiotic or extracts that completely inhibited visible growth of the test organism.

### 3.6. Antibiotic-Extract Combination Experiment (The Time-Kill Method)

The effect of combinations of the methanolic extract of *A. africana* stem bark and selected antibiotics was evaluated using the time-kill assay method in accordance with the descriptions of [13,14]. The extracts and antibiotics were incorporated into 50 mL of nutrient broth at 0.5 × MIC and 1 × MIC, respectively. Controls consisting of nutrient broth incorporated with the extract and the respective antibiotic without the test organism at the test concentrations were included in each experiment. The test and control flasks were inoculated with each test organism to a final inoculum density of approximately 10^5^ cfu/mL. Immediately after inoculation, aliquots (100 μL) of the negative control flasks were taken, serially diluted in sterile physiological saline and plated on nutrient agar in order to determine the zero hour counts. The test flasks were incubated at 37 °C with shaking at 120 rpm. After 24 h of incubation, samples were taken from control and each test flask. The samples from the test flask were transferred to a recovery medium containing 3% Tween 80 to neutralize the effects of the crude extract and antibiotic carry-overs from the test suspensions. Both samples from the recovery medium and the control flasks were then serially diluted in sterile physiological saline. This was later plated on nutrient agar in duplicates. For a better visual observation of the colonies on the agar, 1 mL of 0.5% aqueous solution of 2,3,5 triphenol tetrazolium chloride [15] was added to 100 mL of the molten agar before plating. The plates were incubated at 37 °C for 24 h under aerobic conditions. After incubation, the numbers of colonies were enumerated and the mean counts (cfu/mL) for each test and controls were determined and expressed as log_10_. The interactions were considered synergistic if there was a decrease of ≥2 log_10_ cfu/mL in colony counts after 24 h by the combination compared to the most active single agent [16]. Additivity or indifference was described as a <2 log_10_ cfu/mL change in the average viable counts after 24 h for the combination, in comparison to the most active single drug. Antagonism was defined as a ≥2 log_10_ cfu/mL increase in colony counts after 24 h by the combination compared to that by the most active single agent alone [16,17].

### 3.7. Statistical Analysis

Data were expressed as means ± SD (standard deviation) of five replicates and were statistically analyzed using one way analysis of variance (ANOVA). Means were separated by the Duncan multiple test using Statistical Analysis Software (SAS). Values were considered significant at *P* < 0.05.

## 4. Conclusions

Empiric combination antimicrobial therapy is usually applied to expand antibacterial spectrum and reduce the selection of resistant mutants during treatment. In addition, combinations of agents that exhibit synergy or partial synergy could potentially improve the outcome for patients with difficult to treat infections [28]. Combinational antibiotic therapy to control infections is a viable approach, but will not be effective for a long period of time because of the possible alteration in the susceptibility of bacteria [29]. Therefore, the development of new classes of antimicrobial compounds is of significant importance. One possible approach is to determine whether bioactive compounds from natural products and traditional medicinal plants, which have strong bactericidal activity against pathogenic microorganisms, either show synergistic interaction with antibiotics or enhance the susceptibility level of resistant strains to antibiotics. The methanolic extract from *A. africana* stem bark showed potentials of synergy in combination with some antibiotics against strains of pathogenic organisms that often-present problems of drug resistance. The detection of synergy between crude extract of *A. africana* stem bark and antibiotics demonstrates the potential of this plant as a source of antibiotic resistance modifying compounds. It is necessary to carry out a bioassay guided fractionation of the extract in a bid to isolate and identify the compounds responsible for the synergistic activity with antibiotics. An elucidation of the mechanisms of action of these compounds must be followed by toxicity and *in vivo* tests to determine the therapeutic applicability of such compounds in combination therapy. These are subjects of on-going investigation in our research group.

## Figures and Tables

**Table 1:** Sensitivity patterns of zones of inhibition exhibited by *A. africana* crude methanol stem bark extract against the bacterial isolates Bacterial isolates.

Bacterial isolates	*A. africana* extract (5 mg/mL)	Tetracycline (0.1 mg/mL)	Ampicilin (0.01 mg/mL)
*Enterococcus faecalis* (ATCC 29212)	21 ± 0.0 ^a^	19 ± 0.4 ^a^	17 ± 0.4 ^a^
*Staphylococcus aureus* (ATCC 6538)	13 ± 2.1 ^a^	20 ± 1.0 ^b^	24 ± 0.3 ^b^
*Bacillus pumilus* (ATCC 14884)	19 ± 0.1 ^a^	28 ± 0.0 ^b^	23 ± 1.6 ^a^
*Klebsiella pneumoniae* (ATCC 10031)	16 ± 0.6 ^a^	23 ± 1.3 ^b^	14 ± 0.0 ^a^
*Proteus vulgaris* (CSIR 0030)	20 ± 0.7 ^c^	14 ± 1.7 ^a^	15 ± 1.9 ^b^
*Micrococcus kristinae*§	25 ± 0.9 ^a^	18 ± 0.6 ^b^	18 ± 2.0 ^b^
*Micrococcus luteus*§	23 ± 0.3 ^c^	17 ± 1.2 ^b^	13 ± 1.6 ^a^
*Proteus vulgaris*§	21 ± 1.3 ^a^	22 ± 0.0 ^a^	19 ± 0.0 ^a^
*Klebsiella pneumoniae*§	18 ± 1.6 ^a^	15 ± 0.9 ^a^	13 ± 1.0 ^a^
*Bacillus subtilis*§	20 ± 0.9 ^b^	22 ± 0.6 ^b^	30 ± 0.9 ^a^
*Staphylococcus epidermidis*§	14 ± 1.7 ^b^	10 ± 0.6 ^a^	17 ± 0.2 ^c^

§ Environmental strain;

* Mean of five replicates; Values followed by the same letter along the rows are not significantly different (*P* > 0.05).

**Table 2:** The minimum inhibitory concentrations (MICs) of the extract and the antibiotics used.

	Minimum Inhibitory Concentration (mg/mL)								
Bacterial isolates	Extract	TET	PEN G	ERY	AMX	CIP	CHL	OXT	AMP
*E. faecalis* (ATCC 29212)	0.5	0.016	0.400	0.256	0.412	0.004	0.128	0.008	0.004
*S. aureus* (ATCC 6538)	2.5	0.004	0.001	0.008	0.002	0.004	0.002	0.004	0.001
*B. pumilus* (ATCC 14884)	2.5	0.001	0.001	0.002	0.001	0.002	0.004	0.002	0.001
*K. pneumoniae* (ATCC 10031)	0.1	0.016	0.412	0.256	0.412	0.004	0.256	0.016	0.004
*P. vulgaris* (CSIR 0030)	5.0	0.016	0.032	0.412	0.002	0.004	0.008	0.032	0.001
*M. kristinae*§	0.5	0.004	0.001	0.032	0.001	0.002	0.004	0.004	0.001
*M. luteus*§	0.5	0.004	0.001	0.004	0.008	0.002	0.004	0.004	0.002
*P. vulgaris*§	0.1	0.032	0.004	0.412	0.001	0.001	0.008	0.064	0.004
*K. pneumoniae*§	5.0	0.016	0.412	0.412	0.412	0.001	0.064	0.016	0.004
*B. subtilis*§	1.0	0.001	0.001	0.001	0.001	0.001	0.002	0.004	0.001
*S. epidermidis*§	2.5	0.016	0.412	0.412	0.412	0.001	0.064	0.016	0.004

§ Environmental strain; TET, tetracycline; PEN G, penicillin G; ERY, erythromycin; AMX, amoxicillin; CIP, ciprofloxacin; CHL, chloramphenicol; OXT, oxytetracycline; AMP, ampicillin; Extract, crude methanol extract of *A. Afzelia.*

**Table 3:** *In vitro* activity of Extract-Antibiotic combination at MIC level against test bacterial isolates.

Reduction in bacterial counts (log10 CFU/mL ± SD) ** compared with the two antimicrobial agents used alone								
Bacterial isolates	TET + Extract	PEN G + Extract	ERY + Extract	AMX + Extract	CIP + Extract	CHL + Extract	OXT + Extract	AMP + Extract
*E. faecalis* (ATCC 29212)	−2.0 ± 0.6 (S)	−2.7 ± 0.7 (S)	−2.7 ± 0.6 (S)	−2.8 ± 0.3 (S)	−4.22 ± 0.6 (S)	−2.0 ± 0.06 (S)	−2.04 ± 0.01 (S)	−2.04 ± 0.07 (S)
*S. aureus* (ATCC 6538)	−2.0 ± 0.6 (S)	−2.1 ± 0.3 (S)	−2.04 ± 0.01 (S)	−2.9 ± 1.1 (S)	−2.4 ± 0.04 (S)	−2.0 ± 0.66 (S)	0.52 ± 0.01 (I)	−2.9 ± 0.41 (S)
*B. pumilus* (ATCC 14884)	−2.12 ± 0.6 (S)	−3.1 ± 0.8 (S)	−2.99 ± 1.06 (S)	0.0 ± 0.0 (I)	−3.88 ± 0.5 (S)	−1.99 ± 0.04 (I)	−4.05 ± 1 (S)	−2.7 ± 0.3 (S)
*K. pneumoniae* (ATCC 10031)	0.0 ± 0.0 (I)	−1.99 ± 0.6 (I)	0.0 ± 0.0 (I)	0.5 ± 0.01 (I)	−2.6 ± 0.41 (S)	−3.0 ± 0.21 (S)	−1.92 ± 0.01 (I)	−1.91 ± 1.07 (I)
*P. vulgaris* (CSIR 0030)	−2.04 ± 0.1 (S)	−2.7 ± 0.1 (S)	−4.02 ± 0.9 (S)	0.0 ± 0.0 (I)	−2.0 ± 0.25 (S)	−2.0 ± 0.6 (S)	−2.84 ± 0.26 (S)	−2.85 ± 0.01 (S)
*M. kristinae*§	0.0 ± 0.0 (I)	−2.8 ± 0.9(S)	0.0 ± 0.0 (I)	0.0 ± 0.0 (I)	−3.1 ± 0.04 (S)	−4.22 ± 0.6 (S)	0.0 ± 0.0 (I)	−3.18 ± 2.04 (S)
*M. luteus*§	−2.0 ± 0.6 (S)	−3.8 ± 0.3 (S)	−3.04 ± 0.01 (S)	−2.9 ± 0.9 (S)	−2.1 ± 0.11 (S)	−4.52 ± 0.09 (S)	−3.0 ± 1.49 (S)	−2.89 ± 0.03 (S)
*P. vulgaris*§	0.0 ± 0.0 (I)	−2.4 ± 0.6 (S)	0.0 ± 0.0 (I)	0.2 ± 0.31 (I)	−2.87 ± 0.9 (S)	−4.29 ± 2.6 (S)	0.2 ± 0.4 (I)	−3.87 ± 0.63 (S)
*K. pneumoniae*§	0.0 ± 0.0 (I)	−2.4 ± 0.2 (S)	−2.44 ± 0.11 (S)	0.0 ± 0.0 (I)	−2.65 ± 0.4 (S)	−2.22 ± 1.8 (S)	0.0 ± 0.0 (I)	−2.0 ± 0.11 (S)
*B. subtilis*§	−1.99 ± 0.06 (I)	−1.9 ± 1.92 (I)	−4.22 ± 0.6 (S)	−1.2 ± 0.1 (I)	−2.99 ± 1.7 (S)	−3.22 ± 1.6 (S)	−1.99 ± 0.17 (I)	−2.7 ± 0.22 (S)
*S. epidermidis*§	−1.91 ± 0.07 (I)	−2.8 ± 0.6 (S)	−2.4 ± 0.1 (S)	−1.9 ± 0.1 (I)	−4.01 ± 0.9 (S)	−4.22 ± 0.4 (S)	0.55 ± 1.07 (I)	−1.69 ± 0.06 (I)

§ Note: Environmental strain; TET, tetracycline; PEN G, penicillin G; ERY, erythromycin; AMX, amoxicillin; CIP, ciprofloxacin; CHL, chloramphenicol; OXT, oxytetracycline; AMP, ampicillin; Extract, crude methanol extract of *A. Afzelia*; S, synergy; I, indifference;

** Mean of five replicates.

**Table 4:** *In vitro* activity of Extract-Antibiotic combination at ½ × MIC level against test bacterial isolates.

Reduction in bacterial counts (log_10_ CFU/mL ± SD)** compared with the two antimicrobial agents used alone								
Bacterial isolates	TET + Extract	PEN G + Extract	ERY + Extract	AMX + Extract	CIP + Extract	CHL + Extract	OXT + Extract	AMP + Extract
*E. faecalis* (ATCC 29212)	−2.2 ± 1.9 (S)	−2.0 ± 0.1 (S)	−2.0 ± 1.2 (S)	0.2 ± 0.31 (I)	−2.03 ± 1.1 (S)	−3.2 ± 1.7 (S)	−2.9 ± 1.12 (S)	−2.0 ± 0.61 (S)
*S. aureus* (ATCC 6538)	0.0 ± 0.0 (I)	−3.11 ± 1.6 (S)	−2.0 ± 0.8 (S)	−1.6 ± 0.4 (I)	−3.1 ± 1.9 (S)	−2.0 ± 1.01 (S)	0.0 ± 0.0 (I)	−2.0 ± 1.71 (S)
*B. pumilus* (ATCC 14884)	−3.0 ± 0.11 (S)	−2.6 ± 1.1 (S)	−1.9 ± 0.02 (I)	0.0 ± 0.0 (I)	−2.14 ± 1.2 (S)	−1.0 ± 1.22 (I)	−2.39 ± 0.11 (S)	−2.0 ± 0.61 (S)
*K. pneumoniae* (ATCC 10031)	0.0 ± 0.0 (I)	0.0 ± 0.0 (I)	0.0 ± 0.0 (I)	−1.9 ± 0.6 (I)	−3.1 ± 0.11 (S)	−2.99 ± 0.6 (S)	−0.0 ± 0.0 (I)	0.0 ± 0.0 (I)
*P. vulgaris* (CSIR 0030)	−4.12 ± 1.3 (S)	−2.0 ± 0.9 (S)	−3.8 ± 1.2 (S)	0.0 ± 0.0 (I)	−2.2 ± 1.13 (S)	−2.0 ± 1.6 (S)	−3.04 ± 1.7 (S)	−3.11 ± 1.09 (S)
*M. kristinae*§	−1.20 ± 1.6 (I)	−2.0 ± 1.4(S)	0.0 ± 0.0 (I)	0.0 ± 0.0 (I)	−2.8 ± 1.3 (S)	−2.09 ± 0.1 (S)	0.6 ± 1.1 (I)	−2.18 ± 0.06 (S)
*M. luteus*§	−2.9 ± 1.12 (S)	−2.0 ± 0.61 (S)	−2.1 ± 1.2 (S)	−1.9 ± 1.2 (I)	−2.5 ± 0.15 (S)	−2.44 ± 0.1 (S)	−2.4 ± 0.2 (S)	0.0 ± 0.0 (I)
*P. vulgaris*§	0.0 ± 0.0 (I)	−2.03 ± 1.1 (S)	0.0 ± 0.0 (I)	−0.9 ± 1.2 (I)	−2.03 ± 1.1 (S)	−2.07 ± 1.3 (S)	0.0 ± 0.0 (I)	−1.62 ± 1.5 (I)
*K. pneumoniae*§	0.0 ± 0.0 (I)	−2.0 ± 0.8 (S)	−2.3 ± 1.1 (S)	0.0 ± 0.0 (I)	−2.03 ± 1.1 (S)	−2.0 ± 0.11 (S)	−1.9 ± 1.92 (I)	−2.6 ± 1.07 (S)
*B. subtilis*§	0.0 ± 0.0 (I)	0.0 ± 0.0 (I)	−3.3 ± 0.9 (S)	0.0 ± 0.0 (I)	−2.0 ± 1.1 (S)	−2.62 ± 1.2 (S)	−1.99 ± 0.6 (I)	−2.0 ± 1.9 (S)
*S. epidermidis*§	−1.9 ± 0.02 (I)	−2.2 ± 1.9 (S)	−2.4 ± 0.1 (S)	−1.9 ± 0.7 (I)	−4.12 ± 0.1 (S)	−3.01 ± 1.0 (S)	0.0 ± 0.0 (I)	0.0 ± 0.0 (I)

§ Note: Environmental strain; TET, tetracycline; PEN G, penicillin G; ERY, erythromycin; AMX, amoxicillin; CIP, ciprofloxacin; CHL, chloramphenicol; OXT, oxytetracycline; AMP, ampicillin; Extract, crude methanol extract of *A. Afzelia*; S, synergy; I, indifference;

** Mean of five replicates.
